# Recurrent somatic mutations of *PRKAR1A* in isolated cardiac myxoma

**DOI:** 10.18632/oncotarget.21916

**Published:** 2017-10-19

**Authors:** Jian He, Mingju Sun, Enyou Li, Yingyong Hou, Matthew J. Shepard, Di Chen, Karel Pacak, Changsong Wang, Lei Guo, Zhengping Zhuang, Yang Liu

**Affiliations:** ^1^ Scientific Research Center for Translational Medicine, Department of Biotechnology, Dalian Institute of Chemical Physics, Chinese Academy of Sciences, Dalian, China; ^2^ Department of Anesthesiology, First Affiliated Hospital of Harbin Medical University, Harbin, China; ^3^ Surgical Neurology Branch, National Institute of Neurological Disorders and Stroke, National Institutes of Health, Bethesda, Maryland; ^4^ Department of Critical Care Medicine, The Third Affiliated Hospital of Harbin Medical University, Nangang District, Harbin, China; ^5^ Department of Pathology, School of Basic Medical Sciences & Zhongshan Hospital, Fudan University, Shanghai, China; ^6^ Department of Neurologic Surgery, University of Virginia Health System, Charlottesville, Virginia, USA; ^7^ Eunice Kennedy Shriver National Institute of Child Health and Human Development, National Institutes of Health, Bethesda, Maryland, USA

**Keywords:** cardiac myxomas, PRKAR1A, somatic mutation

## Abstract

**Background:**

Cardiac myxomas are benign tumors that commonly arise within the left atria. Familial cardiac myxomas are a part of Carney Complex (CNC), an autosomal dominant multiple neoplasia syndrome caused by germline mutations in *PRKAR1A*. Seven percent of cardiac myxomas are associated with CNC. To date, the genetic basis of isolated cardiac myxomas (ICM), however, has not been fully elucidated.

**Methods:**

We investigated the genetic profile of ICM using whole exome sequencing (WES). Suspected mutations were confirmed using targeted sanger sequencing. To further examine the presence of *PRKAR1A* mutations in ICM, we performed targeted sequencing in an additional 61 ICM specimens.

**Results:**

87.5% (7/8) of ICM harbored mutations in *PRKAR1A*. Three of the 8 ICM harbored biallelic somatic mutations of *PRKAR1A*, including c.607_610del:p.Leu203fs (pathogenic) + c.C896G:p.Ser299X (pathogenic), c.952delT:p.Leu318fs (pathogenic) + c.769-2 A>G (pathogenic) and c.178-1 G>C (pathogenic) + c. 550+1 G>C (pathogenic). Four of 8 tumors harbored monoallelic *PRKAR1A* mutations, including c.523_524insG:p.Tyr175_Val176delinsX (pathogenic), c.C920A:p.Ser307X (pathogenic), c.30delG:p.Glu10fs (pathogenic) and c.C289T:p.Arg97X (pathogenic). No identical variants were observed across the 8 ICM samples. Interestingly, none of these variants have been previously described in familial cardiac myxomas. In order to confirm our findings, directed sequencing of 61 ICM specimens was subsequently performed. Sixty-four percent (39/61) of ICMs tumors contained inactivating *PRKAR1A* mutations.

**Conclusion:**

Our findings suggest that loss-of-function mutations of *PRKAR1A* may play a vital role in the formation of isolated cardiac myxomas.

## INTRODUCTION

Cardiac myxomas (CMs) are benign cardiac tumors that are characterized by stellate to plump, cytologically bland, mesenchymal cells situated in a myxoid stroma [1, 2]. These tumors often arise as a solitary mass situated in the left atria and have been described to occur more commonly in women than men [3, 4]. Isolated cardiac myxomas (ICM) tend to occur in individuals in the sixth to seventh decade of life and can present with syncope, palpitations, dyspnea or heart failure [5]. Approximately 7% of CMs occur in association with carney complex (CNC), a tumor predisposition syndrome characterized by the development of pigmented cutaneous lesions, myxomas and multiple endocrine neoplasms [6]. Investigation of the pathogenesis of CNC revealed that 70% of affected individuals harbored germline mutations in *PRKAR1A,* a gene that encodes the regulatory subunit (type I-alpha) of protein kinase A (PKA) [7-10]. Indeed, *PRKAR1A* mutations have been described in two-thirds of CNC-associated CMs. Loss of function mutations in *PRKAR1A* lead to increased PKA activation thereby promoting CREB phosphorylation and upregulation of the MAPK and Rb/E25 signaling pathways. This dysregulation is believed to play an important role in tumorigenesis [11]. Genetic aberrations of *PRKAR1A* have been investigated in ICM [12-14]. Several authors have reported that ICMs do not harbor *PRKAR1A* mutations and have suggested that the genetic basis of ICM may differ from CNC associated CMs [13, 14]. In contradistinction, a recent study suggested that 31% of ICM may contain inactivating mutations in *PRKAR1A* [5]. Despite this report, whether *PRKAR1A* mutations are involved in the pathogenesis of ICM remains controversial and a subject of ongoing debate.

## RESULTS AND DISCUSSION

The development of next generation sequencing techniques has facilitated the discovery of previously unidentified cancer driver mutations [15]. We speculated that the discrepancy in the literature regarding the presence of *PRKAR1A* mutations in ICM could be due to lack of depth and coverage of previously utilized DNA sequencing methods.

**Figure 1:**
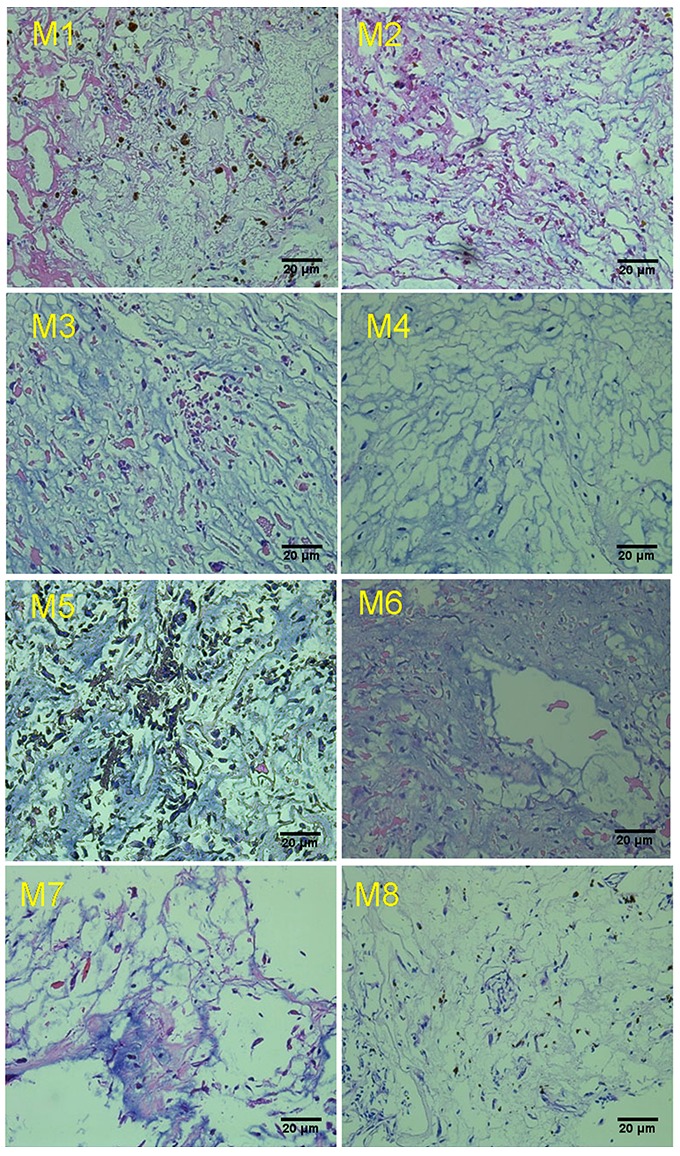
Representative Hematoxylin and Eosin staining of cardiac myxoma tissue M1 to M8 indicate the case number from ICM specimens obtained from different patients.

In this study, whole exome sequencing (WES) was performed on 8 cardiac myxoma specimens derived from patients without any other manifestation of CNC. The median age of the patients at diagnosis was 51 years (range 44-60 years). Six of the 8 (75%) patients were female. All of the tumors arose within the left atrium, consistent with previous reports [16]. The pathological phenotype of these primary ICM tissues was confirmed by hematoxylin and eosin (H&E) staining (Figure 1).

**Figure 2:**
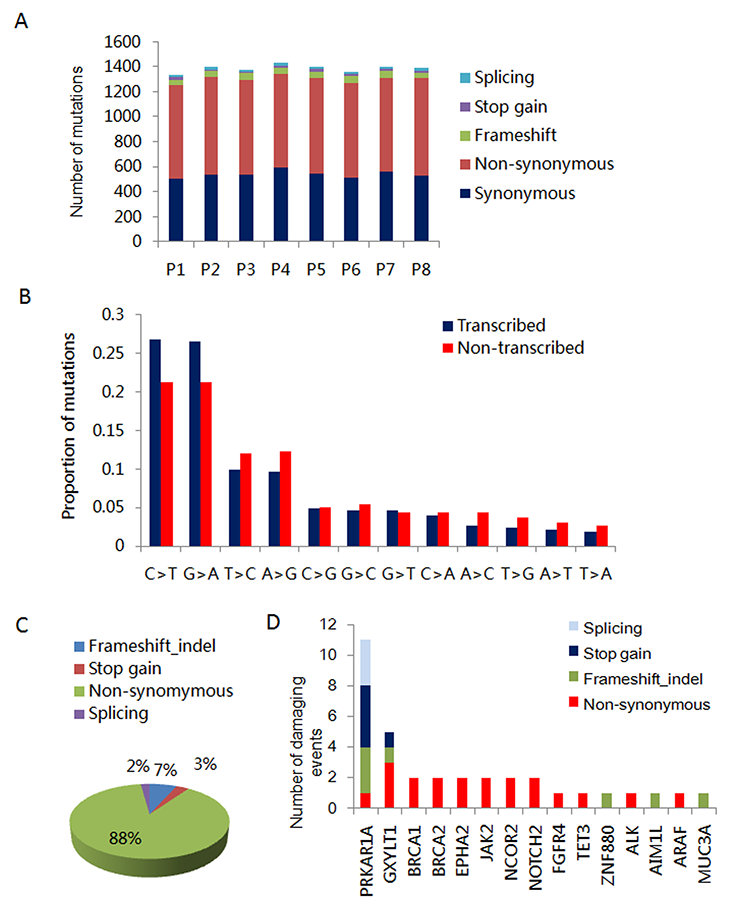
Inactivating mutations of PRKARR1A in ICM **(A)** Number of splicing, stop-gain, frameshift, synonymous and non-synonymous mutations in the 8 ICM samples. **(B)** Relative proportions of base-pair substitutions on the transcribed and non-transcribed strands among the 616 point mutations identified in the exome sequences of 8 ICMs. **(C)** Percentage of differential types of variants identified by WES in 8 ICMs. **(D)** Number and type of damaging events for tumor associated genes altered in 8 ICMs.

To explore the genetic variation in ICM, we performed whole exome sequencing as previously described [17]. The details of capture statistics and coverage are presented in Supplementary Table 1. After barcode-based sample deconvolution, sequence reads were mapped with BWA tools to the human genome (hg19). Sequence variant calls were performed by GATK after removing potential PCR duplicates. We called, on average 80,425 base substitutions and 8,061 insertions/deletions per tumor. Missense-, nonsense-, or splice-site-altering variants absent from the 1000 Genomes dataset were selected for further consideration. The majority of the identified variants were observed in the intronic and exonic regions, accounting for 44.81% and 33.77% of identified variants, respectively (Supplementary Table 1). These variants included synonymous, non-synonymous, stop-gain, stop-loss, splice-site and frameshift mutations. The majority of detected mutations were non-synonymous, accounting for 750-800 identified mutations. This is in contrast to the 14-22 splice-site mutations that were identified in the ICM from each patient (Figure 2A). Interestingly, we found that the majority of the base pair changes in the transcribed region were C>T (G>A) substitutions (Figure 2B). To identify pathogenic mutations, we excluded single nucleotide polymorphisms (SNP), synonymous mutations, and variants in the untranslated, intronic and intergenic region. We identified 4391 variants, covering 3198 genes across all eight samples. Mutations that were suspected to result in altered gene product activity accounted for 12% of these variants. These included frameshift, stop gain and splice-site mutations (Figure 2C). Additionally, we found 15 inactivating mutations involving *PRKAR1A*, which have been described in CNC related CM (Figure 2D). Oncogenes such as BRCA1/2, JAK2, NOTCH2, EPHA2 and NCOR2 harbored 2 nonsynonymous mutations, which are extremely rare variants reported in the dbsnp database. Other mutated genes included GXYLT1 (1 frameshift, 1 stop gain and 3 non-synonymous mutations). Furthermore, we found inactivating mutations of ZNF880 and MUC3A in all 8 ICM tissue samples. AIM1L was mutated in 5 of 8 analyzed ICM. However, we found that these variants were germline mutations due to the presence of these mutations in patients matched peripheral blood DNA (data not shown).

The loss-of-function mutations of *PRKAR1A* have been previously identified in CNC and familial CMs. We found 11 inactivating mutations of *PRKAR1A* in 7 of 8 (86%) analyzed ICM tumors (Table 1). 3 of 8 (37.5%) ICM specimens contained biallelic *PRKAR1A* mutations, including c.607_610del:p.Leu203fs (pathogenic) + c.C896G:p.Ser299X (pathogenic), c.952delT:p.Leu318fs (pathogenic) + c.769-2 A>G (pathogenic) and c.178-1 G>C (pathogenic) + c. 550+1 G>C (pathogenic). Four of 8 (50%) tumors harbored heterozygous *PRKAR1A* mutations, including c.523_524insG:p.Tyr175_Val176delinsX (pathogenic), c.C920A:p.Ser307X (pathogenic), c.30delG:p.Glu10fs (pathogenic) and c.C289T:p.Arg97X (pathogenic). In all cases, we could not detect the same genetic alterations in the matched blood samples, suggesting that these variants were somatic mutations (Supplementary Figure 1).

**Table 1:** Somatic mutations of *PRKAR1A* in 8 ICM patients

Sample	Age	Sex	Diagnosis	PRKAR1A mutations	Variant classification
M1	48	F	Myxoma	c.523_524insG:p.Tyr175_Val176delinsX	Pathogenic
M2	46	F	Myxoma	c.C920A:p.Ser307X	Pathogenic
M3	53	F	Myxoma	c.30delG:p.Glu10fs	Pathogenic
M4	46	M	Myxoma	c.952delT:p.Leu318fs	Pathogenic
				c.769-2 A>G	Pathogenic
M5	44	M	Myxoma	c.178-1 G>C	Pathogenic
				c. 550+1 G>A	Pathogenic
M6	53	F	Myxoma	c.607_610del:p.Leu203fs	Pathogenic
				c.C896G:p.Ser299X	Pathogenic
M7	56	F	Myxoma	c.C289T:p.Arg97X	Pathogenic
M8	60	F	Myxoma	Not detected	-

Next, we investigated the effect of these inactivating variants on PRKAR1A expression in primary tumor tissues by immunohistochemistry (IHC). In agreement with the mutation analysis, PRKAR1A expression was not identified in the 7 tumors that contained somatic *PRKAR1A* mutations, suggesting the abrogation of PRKAR1A function in ICM (Supplementary Figure 2). To further assess the *PRKAR1A* mutation in ICM, we investigated the mutation status of *PRKAR1A* in an additional 61 ICM tumors. We amplified the 10 exons of *PRKAR1A* by PCR, and performed targeted Sanger DNA sequencing. In this cohort, 39 of 61 (64%) ICM specimens harbored inactivating *PRKAR1A* mutations (Table 2). Similar to our WES data, no hotspot mutation was found in these samples. In addition, most of the *PRKAR1A* mutations were distributed across Exons 1-5. While expression of PRKAR1A varied across these samples, we speculate that some of them may have resulted in the truncation of the PRKAR1A protein due to the fact that most of the identified mutations were frameshift and nonsense mutations. For instance, case No.9 showed PRKAR1A positive due to the antibody, which may still be able to recognize 200-260 aa of truncated PRKAR1A protein.

**Table 2:** Analysis of *PRKAR1A* mutations in 61 FFPE ICMs by Sanger sequencing. “+” indicates the positive expression of *PRKAR1A* in these FFPE tissue samples, while “-” indicates negative expression of this protein. All the variants were classified according to ACMG criteria

Sample	PRKAR1A mutations	Variant classification	IHC	Sample	PRKAR1A mutations	Variant classification	IHC
1	Not detected		+/-	32	c.421-440del:p.Leu141fs	Pathogenic	-
2	c.8delC:p.Ser3fs	Pathogenic	+	33	Not detected		-
3	c.C73G:p.His25Asp	Likely pathogenic	-	34	Not detected		-
4	c.C205T:p.Gln69X	Pathogenic	-	35	Not detected		-
5	Not detected		+	36	c.8delC:p.Ser3fs	Pathogenic	-
6	c.162delG:p.Glu85fs;162_163insG, p.Glu85fs, c.912_913insT:p.Leu271fs	Pathogenic	-	37	c.290delG:p.Arg97fs	Pathogenic	-
7	Not detected		+	38	Not detected		+
8	c.A1115C:p.Gln372Pro	Likely pathogenic	-	39	c.162delT:p.Phe54fs	Pathogenic	+
9	c.267delA:p.Pro89fs, c.A367T:p.Lys123X	Pathogenic	+	40	Not detected		+
10	c.482delG:p.Gly161fs		-	41	c.478delG:p.Ala160fs	Pathogenic	++
11	c.A1093T:p.Ile365Phe	Likely pathogenic	+/-	42	Not detected		-
12	Not detected		-	43	Not detected		+
13	c.A872G:p.Glu291Gly	Likely pathogenic	+	44	c.C124T:p.Arg42X	Pathogenic	-
14	c.1059delT:p.Pro353fs	Pathogenic	-	45	c.289C>Tp.Arg97X	Pathogenic	-
15	Not detected		+	46	c.1059delT:p.Pro353fs	Pathogenic	-
16	c.421-440del:p.Leu141fs	Pathogenic	+	47	c.T89A:p.Leu30Gln, c.502+2T>G	Pathogenic	+
17	c.767delT:p.Leu256X, c.892-2A>T	Pathogenic	-	48	c.200-213del:p.Asn67fs	Pathogenic	+
18	Not detected		+	49	Not detected		+
19	c.201-213del:p.Asn67fs	Pathogenic	+	50	c.290delG:p.Arg97fs	Pathogenic	-
20	c.219_220insGTAAGGCACT:p.Arg74fs	Pathogenic	+	51	Not detected		+
21	c.421-440del:p.Leu141fs	Pathogenic	-	52	c.10_11insTG:p.Gly4fs	Pathogenic	+
22	Not detected		-	53	Not detected		-
23	c.8delC:p.Ser3fs	Pathogenic	-	54	c.783delG:p.Lys261fs	Pathogenic	+
24	c.550-2A>G	Pathogenic	+	55	c.10_11insGT:p.Gly4fs	Pathogenic	-
25	Not detected		+	56	c.550-2A>G	Pathogenic	+
26	c.G569A:p.Trp190X, c.737-738del:p.Tyr246fs	Pathogenic	-	57	Not detected		-
27	c.453delT:p.DAsp151fs	Pathogenic	-	58	c.478delG:p.Ala160fs, c.T619A:p.Tyr207Asn	Pathogenic	-
28	Not detected		+	59	c.251-300del:p.Pro84fs	Pathogenic	-
29	c.C124T:p.Arg42X	Pathogenic	+	60	c.C196T:p.Gln66X	Pathogenic	+
30	Not detected		+	61	Not detected		+
31	c.349-1G>A	Pathogenic	-				

Overall our results suggest that ICM may contain *PRKARA1A* mutations in approximately two-thirds of cases. The remaining samples may contain mutations at levels below our detection limit due to sample procurement. On the other hand, we speculate that there may be other driver mutations in the non-PRKAR1A mutated ICM specimens that have yet to be identified. Therefore, we examined the whole exome sequencing results from the patient whose ICM did not contain a *PRKAR1A* mutation. In this specimen, we found that the patient presented with a nonsense mutation in *MXI1*, a tumor suppressor gene that has been reported to be frequently inactivated in prostate cancer [18, 19]. This mutation, however, was determined to be a germline mutation, supported by the presence of this mutation in matched blood sample. Interestingly, this patient also carried *NOTCH2*, *EPHA2, NCOR2*, *FGFR4,* and *ARAF* germline missense mutations. These non-SNP variants were not found in the other patients with *PRKAR1A* mutations. Unfortunately, we could not identify any clinical or pathologic differences between *PRKAR1A* mutated and non-mutated ICM patients. Although this is only one sample, this raises the possibility that ICM that do not harbor *PRKAR1A* mutations may be genetically heterogeneous with multiple genetic variants contributing to tumorigenesis. Thus, further work is necessary to define the clinical and pathologic differences between *PRKAR1A-*mutated and *PRKAR1A* non-mutated isolated cardiac myxomas. Our work, reported herein, clearly defines that *PRKAR1A* mutations are present in a substantial proportion of isolated cardiac myxomas.

## MATERIALS AND METHODS

### Ethics statement

Blood and tumor samples from ICM patients with were obtained under the approval of the Ha’er Bin Medical University Internal Review Board. Written informed consent was obtained from all patients.

### Clinical samples

A total of 8 paired ICM tissues and blood samples were recruited from the Affiliated Hospital of Harbin Medical University (Harbin, China). 61 FFPE ICM tissue samples were obtained from Zhongshan Hospital of ShangHai.

### Whole exome sequencing

Genomic DNA was isolated and sequenced using standard protocols for next generation sequencing (Compass Biotechnology, Co. Ltd. Beijing, China). Briefly, the Agilent SureSelect Human All Exon 60 Mb kit (Agilent Technologies, Santa Clara, CA, USA) was utilized to capture all exons. Shotgun libraries were established by shearing DNA and ligating sequencing adaptors bearing a 6 base-pair index, followed by polymerase chain reaction (PCR). Libraries were hybridized to SureSelect Human All Exon V6 solution-based probes, amplified, pooled, and sequenced on Illumina Hiseq X10 (PE 150 bp). The final targeted region was 60,456,963 base pairs, including approximately 20,000 genes (230,418 exons and splice junctions) in the genome. The average mean fold coverage was 203X. 97.9% of target bases were covered at least at once and 97.5% were covered at least 20X.

### Validation of variants by Sanger sequencing

The germline and somatic variants identified by WES were further analyzed by Sanger Sequencing. Briefly, primers were designed by generunner software to amplify the genomic region using the extracted DNA from ICM tissues and blood samples. The PCR product was sent for automatic DNA sequencing.

### Hematoxylin and eosin (H&E) staining

ICM primary tissues were fixed in 4% paraformaldehyde for 24h, washed in PBS and were embedded in paraffin. Two-micrometer sections were stained with hematoxylin and eosin following standard procedures.

### Immunohistochemistry

For immunohistochemistry (IHC), the antigen retrieval step was performed with EDTA Buffer (1mM EDTA, 0.05% Tween 20, pH 8.0) in a pressure cooker. Endogenous peroxide activity was blocked by 3% peroxidase, and the slides were further blocked by 5% BSA to prevent nonspecific binding. Primary antibodies directed against PRKAR1A (Abcam, ab139695) incubated at an optimal dilution (1:100). The secondary antibody was donkey anti-rabbit IgG H&L (Abcam, ab6802, diluation 1:1000). For detection, the Polink-2 Plus horseradish peroxidase (HRP) Polymer Detection system (PV-9001; GBI Labs, Mukilteo, WA, USA) was used. Hematoxylin dye was used as counter stain. The slides were examined with an Olympus BX61 microscope with cell Sens Standard Software Version 1.6 (Olympus Corporation, Tokyo, Japan).

## SUPPLEMENTARY MATERIALS FIGURES AND TABLE


