# Supplementary material for: Recurrent somatic mutations of PRKAR1A in isolated cardiac myxoma
Source: Oncotarget. 2017 Oct 19;8(61):103968–74. doi: 10.18632/oncotarget.21916 (PMC5732779; doi:10.18632/oncotarget.21916)
Supplement: Supplementary file 1 [file oncotarget-08-103968-s001.pdf]

Recurrent somatic mutations of *PRKAR1A* in isolated cardiac myxoma

SUPPLEMENTARY MATERIALS

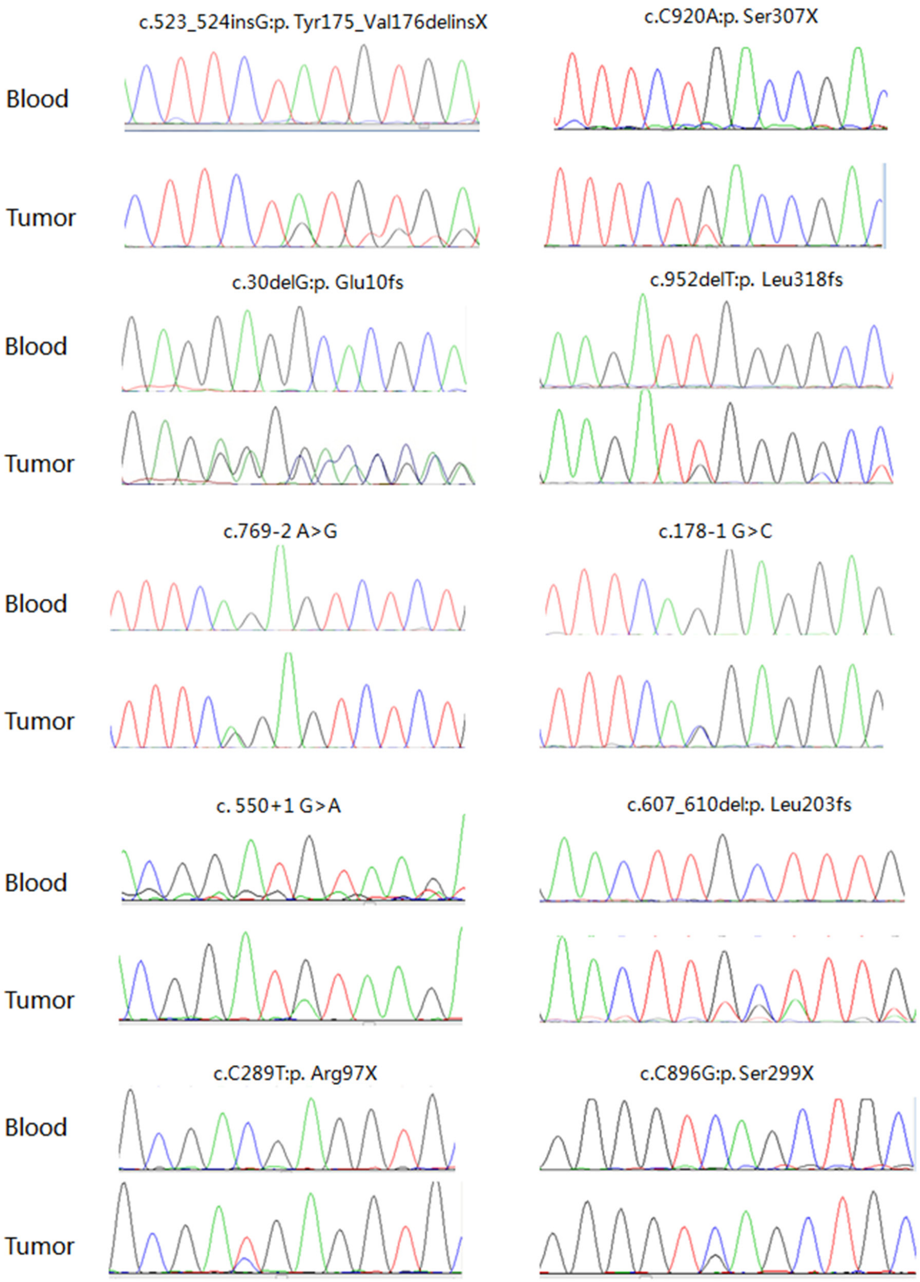

Supplementary Figure 1: Validation of somatic variants identified in 8 ICMs by Sanger Sequencing.

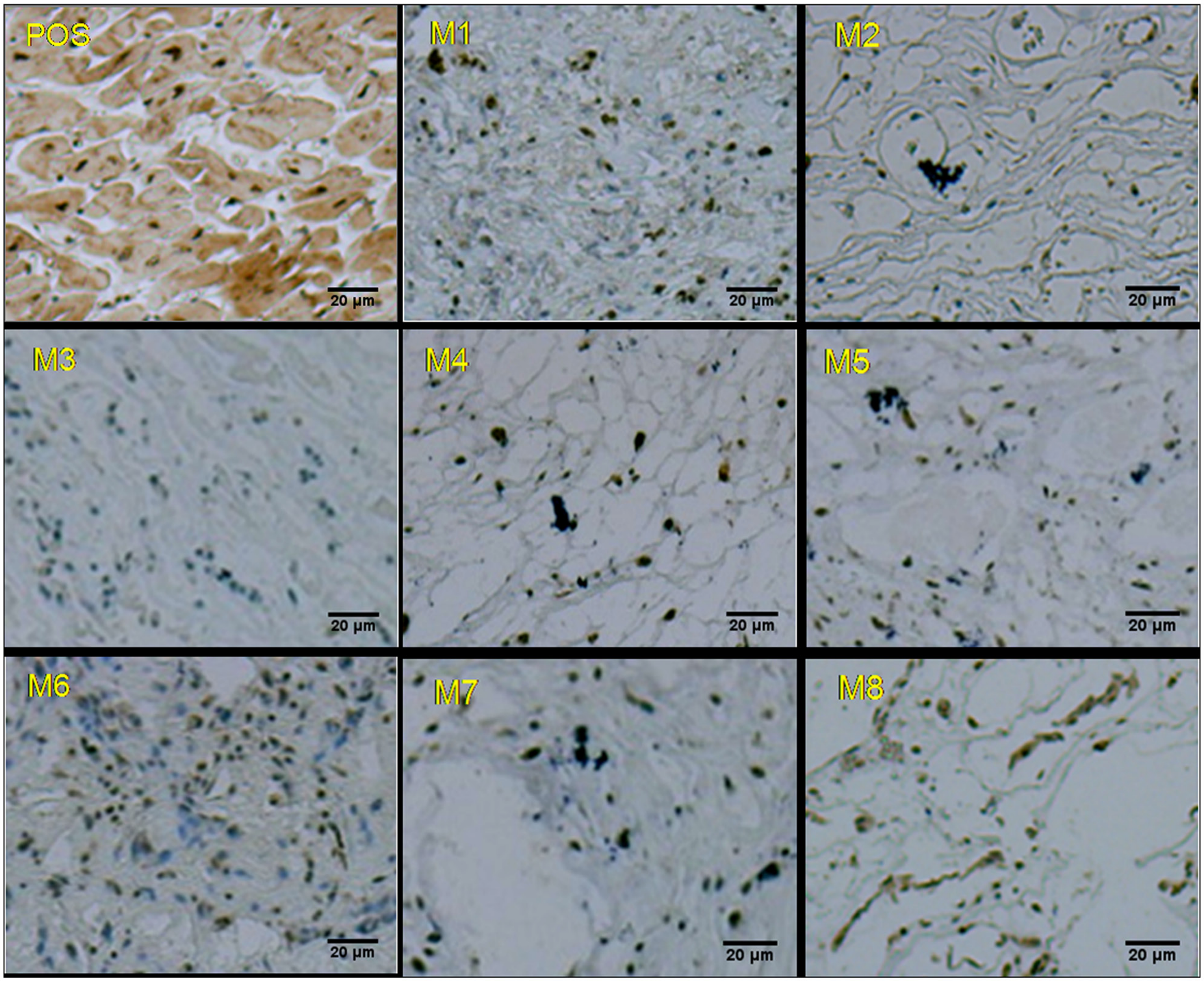

Supplementary Figure 2: Representative photomicrographs of anti-PRKAR1A immunohistochemistry performed on 8 isolated cardiac myxoma specimens that had undergone sequencing.

Supplementary Table 1: Number and percentage of identified variants across the genome region

Type	Count	Percent
DOWNSTREAM	21601	8.98%
EXON	81212	33.77%
INTERGENIC	4094	1.70%
INTRON	98155	40.81%
SPLICE_SITE_ACCEPTOR	249	0.10%
SPLICE_SITE_DONOR	210	0.09%
SPLICE_SITE_REGION	8114	3.37%
TRANSCRIPT	7	0.00%
UPSTREAM	15934	6.63%
UTR_3_PRIME	5599	2.33%
UTR_5_PRIME	5321	2.21%
